# Effect of GLP-1 Receptor Agonists on Patients with Thyroid Carcinomas Undergoing Active Surveillance

**DOI:** 10.1210/jendso/bvaf182

**Published:** 2025-11-14

**Authors:** Armando Patrizio, Samantha K Newman, R Michael Tuttle, Laura Boucai

**Affiliations:** Endocrinology Service, Department of Medicine, Memorial Sloan Kettering Cancer Center, New York, NY 10021, USA; Endocrinology Service, Department of Medicine, Memorial Sloan Kettering Cancer Center, New York, NY 10021, USA; Endocrinology Service, Department of Medicine, Memorial Sloan Kettering Cancer Center, New York, NY 10021, USA; Endocrinology Service, Department of Medicine, Memorial Sloan Kettering Cancer Center, New York, NY 10021, USA

**Keywords:** GLP-1RA, thyroid microcarcinomas, active surveillance

## Abstract

**Context:**

Glucagon-like peptide-1 receptor agonists (GLP-1RAs) have emerged as effective therapies for diabetes mellitus and obesity. Their effect on nonmedullary thyroid malignancies remains unclear.

**Objective:**

To evaluate the impact of GLP-1RA exposure on tumor kinetics of patients with low-risk papillary thyroid carcinoma undergoing active surveillance (AS).

**Design:**

Retrospective observational cohort study of 18 patients with 19 papillary thyroid carcinomas (≤1.5 cm) exposed to GLP-1RA matched 1:2 by body mass index and tumor size to 37 patients with 38 carcinomas never exposed to GLP-1RA and undergoing AS at a single tertiary center for a median of 5.5 years.

**Main outcome:**

Tumor growth/shrinkage was considered significant when any diameter changed by ≥3 mm and/or volume changed >72%. Tumor volume doubling time was calculated in a subset of patients off and on GLP-1RA therapy.

**Results:**

After a median GLP-1RA exposure of 25 months [interquartile range: 14-34] and a median follow-up of 5.5 years, 2/19 (10.5%) tumors exposed to GLP-1RA exhibited significant volume growth, 1 (5.3%) decreased, and 16 (84.2%) remained stable, whereas 1/38 (2.6%) carcinomas not exposed to GLP-1RA showed >72% volume increase, 2/38 (5.3%) decreased, and 35/38 (92.1%) remained stable, *P* = .53. GLP-1RA exposure did not alter tumor volume growth kinetics in either of the 2 tumors that increased over time.

**Conclusion:**

GLP-1RA therapy does not affect tumor growth kinetics in patients with low-risk papillary thyroid carcinoma on AS. Further studies with larger cohorts and extended follow-up are warranted to validate the safety of GLP-1RA use in patients with thyroid carcinomas undergoing active surveillance.

Glucagon-like peptide-1 receptor agonists (GLP-1RAs) have significantly advanced the treatment of diabetes and obesity along with their associated cardiometabolic complications since their initial approval by the U.S. Food and Drug Administration (FDA) in 2005 [1, 2]. With the global rise in these conditions, the number of patients who may benefit from GLP-1RA therapy is expected to grow substantially in the near future [3, 4].

Although GLP-1RAs generally have a favorable safety profile, concerns have emerged regarding a potential link to medullary thyroid cancer (MTC). Rodent studies have shown that GLP-1RA stimulates calcitonin production and growth of thyroid parafollicular C cells and MTC in a dose- and drug-duration dependent manner [5, 6].This has led the FDA to issue a black box warning against their use in individuals with a personal or family history of MTC. However, these effects have not been observed in primates or humans, and clinical data to date have not shown a notable rise in MTC cases among GLP-1RA users [7, 8].

GLP-1 receptor expression has been detected in human thyroid follicular cells and certain papillary thyroid cancers (PTC) [9, 10], prompting further investigation into whether these drugs may promote growth of follicular-cell derived differentiated thyroid cancer. To date, the association between GLP-1RA and thyroid cancer remains inconclusive. In a case-control study from France, Bezin et al compared 2562 patients with type 2 diabetes mellitus and thyroid cancer exposed to GLP-1RA to 45 184 controls matched by age, sex, and duration of diabetes and found that GLP-1RA users had an increased risk of thyroid cancer compared to nonusers [11]. Real-world and pharmacovigilance studies have also indicated a higher detection signal for differentiated thyroid cancer [12, 13]. On the other hand, a Scandinavian study and an international cohort of 145 410 and 98 147 GLP-1 users, respectively, have not found a significant association between GLP-1RAs use and thyroid cancer risk [14, 15]. Additionally, long-term follow-up of randomized trials and meta-analyses have not reported an increased incidence of neoplasms, including thyroid cancer, in patients treated with GLP-1RAs [16, 17]. More recently, a retrospective emulation of an idealized target trial involving 351 913 patients with type 2 diabetes found an increased risk of thyroid cancer diagnosis among GLP-1RA users only in the first year of treatment, suggesting that this increased risk may be attributed to increased vigilance and case detection rather than to carcinogenic effects of the drug [18]. In the absence of conclusive evidence regarding the potential association between GLP-1RA exposure and thyroid cancer risk, both patients and health care providers are often required to make therapeutic decisions in a context of uncertainty—particularly in individuals with preexisting thyroid nodules or a prior diagnosis of differentiated thyroid carcinoma.

Active surveillance of low-risk PTC has been recognized by the ATA and NCCN guidelines as an alternative approach to immediate surgery given long-term stability of these small tumors and overall safety of a delayed intervention [19, 20]. Many patients with thyroid carcinomas under active surveillance suffer from obesity and diabetes that, if not treated adequately, could result in more detrimental complications than the hypothetical minor growth risk of microcarcinomas exposed to GLP-1RA.

Given the lack of data on the impact of GLP-1RA in patients with thyroid carcinomas, the present study aims to determine, for the first time, the effect of GLP-1RA on tumor growth and progression of low-risk papillary thyroid carcinomas undergoing active surveillance.

## Materials and Methods

### Study Design and Population

This is a retrospective observational cohort study of patients with low-risk thyroid carcinomas undergoing active surveillance and taking GLP-1RA, matched 1:2 by body mass index (BMI) and tumor size to a cohort of patients never exposed to GLP-1RA and followed at a single tertiary care referral institution (Memorial Sloan Kettering Cancer Center [MSKCC], New York, New York). Of 441 patients with low-risk thyroid carcinomas on active surveillance, 22 were exposed to GLP-1RA.

Inclusion criteria for active surveillance at MSKCC have been previously described [21]. These consist of a diagnosis of PTC (Bethesda VI) or suspicious for PTC (Bethesda V) based on cytologic, molecular, and ultrasonographic findings confirmed at MSKCC with a maximal tumor diameter at baseline ≤1.5 cm and no clinical or radiographic evidence of extrathyroidal extension, invasion of local structures, or regional/distant metastases [21].

Additional criteria for inclusion in this study were (1) follow-up by an endocrinologist and/or head and neck surgeon at MSKCC for ≥6 months; (2) neck ultrasonography performed at MSKCC at 6-month intervals for the first 2 years, yearly for the next 3 years, and then approximately every 2 years for ongoing follow-up; and (3) exposure to GLP-1 RA for at least 6 months. Levothyroxine was prescribed to patients as needed to maintain a TSH level <3 mIU/L while avoiding TSH suppression.

Of the 22 patients identified with thyroid carcinomas and taking GLP-1RA, 4 were excluded: 1 patient had GLP-1RA exposure for less than 6 months and 3 patients did not have ultrasound surveillance since initiation of GLP-1RA therapy. The final cohort included 19 low-risk papillary thyroid carcinomas ≤1.5 cm in size from 18 adult patients exposed to GLP-1RA therapy for either diabetes mellitus or obesity and followed with active surveillance. These were matched 1:2 by BMI and by tumor size to a cohort of 37 patients with 38 low-risk thyroid carcinomas never exposed to GLP-1RA and undergoing active surveillance at MSKCC.

### Variables

The following patient variables were extracted by reviewing each patient's medical chart: age at time of PTC diagnosis, sex, race and ethnicity, diabetes mellitus status, weight, BMI, hemoglobin A1c at PTC diagnosis and at last follow-up, and PTC ultrasonographic diameters and volumes. The volume (mL) of the tumor was calculated using the ellipsoid volume formula: π/6 · length · width · height. Follow-up was defined as the time from PTC diagnosis until the last thyroid ultrasound. If diagnosis of PTC was done outside of MSKCC, we required that every patient had surveillance ultrasounds with Memorial Sloan Kettering Cancer Center radiologists.

### Exposure to GLP1-RAs

With the aim of investigating the drug class effect on PTC biology, the GLP-1RA exposure time was collected, considering all FDA-approved molecules available in the United States (dulaglutide, exenatide, liraglutide, lixisenatide, semaglutide, and tirzepatide), regardless of administration route (injectable or oral). The exposure time was calculated using the date of the first filled prescription as the start date, and the discontinuation date (or the last follow-up date if the prescription was still active) as the end date, as recorded in the MSKCC electronic medical record. In 6 patients, the date of GLP-1RA initiation preceded the thyroid carcinoma diagnosis date and patients continued GLP-1RA treatment during the active surveillance of their thyroid cancer.

### Outcomes and Statistical Analysis

Tumor growth/shrinkage and tumor volume kinetics were compared in patients exposed and never exposed to GLP-1RA. Additionally, tumor growth and volume kinetics were examined within patients exposed to GLP-1RA when they were off and on the drug. Continuous data are presented as means with SDs or medians with interquartile ranges for nonparametric variables. Meaningful tumor growth was defined as a 3-mm increase or more in any diameter and/or >72% volume increase compared to baseline. For tumor volume doubling time to be calculated, at least 3 ultrasound measurements were needed for each nodule on and off the drug. Tumor volume doubling time (TVDT) was calculated by fitting a least squares regression model to log-transformed tumor volume measurements and plotted on semi-log curves using the Kuma hospital calculator [22]. Meaningful tumor shrinkage was defined as a 3-mm decrease or more in any diameter and/or >72% volume decrease compared to maximum diameter or baseline tumor volume [23]. Chi-squared or Fisher exact test (when appropriate) were used to compare proportions between cohorts exposed and not exposed to GLP-1RA. Spearman correlation was used to test correlation between duration of GLP-1RA use or weight loss and maximal volume change. A *P* value <.05 was considered statistically significant. All analyses were performed using STATA 17 (StataCorp LLC, College Station, TX).

### Study Approval

The protocol was approved by the MSK Institutional Review Board (# 16-1400) and Privacy Board-B and the study was performed in accordance with the Declaration of Helsinki as revised in 2013.

## Results

A total of 18 patients with 19 low-risk thyroid carcinomas exposed to GLP-1RA were followed for a median of 66 months and matched 1:2 by BMI and tumor size to a cohort of 37 patients with 38 low-risk thyroid carcinomas never exposed to GLP-1RA. Baseline demographic, clinical, and pathological characteristics of our cohorts are summarized in Table 1. There was no difference in the median age at diagnosis (58 years; interquartile range [IQR] 53-67 vs 64 years [IQR: 53-73], *P* = .201), sex (females: 58% vs 63%, *P* = .7), race (white: 84% vs 95%, *P* = .14), median body mass index (BMI: 30.1 kg/m^2^ [IQR: 26.5-33.9] vs 29.43 kg/m^2^ [IQR: 25.5-35.5], *P* = .52), or median tumor size (0.8 cm [IQR:0.6-1.3 cm] vs 0.9 cm [IQR: 0.7-1.1 cm], *P* = .876) between patients exposed and not exposed to GLP-1RA. A similar proportion of tumors were diagnosed as Bethesda V according to the 3rd edition of The Bethesda System for Reporting Thyroid Cytopathology [24], 6/19 (32%) vs 8/38 (21%), or Bethesda VI 13/19 (68%) vs 30/38(79%), *P* = .384 comparing the cohort exposed and not exposed to GLP-1RA. More patients exposed to GLP-1RA had a diagnosis of diabetes 11/18 (61%) vs 9/37 (24%), *P* = .008. (Table 1).

**Table 1.** Clinicopathological features of the study cohort

Clinical characteristics	Exposed to GLP-1RA (patients n = 18, carcinomas n = 19)	Not exposed to GLP-1RA (patients = 37, carcinomas n = 38)	*P* value
**Age at diagnosis (years)**			
Median [IQR]	58 [53-67]	64 [53-73]	.201
**Sex**			
Female	11 (58)	24 (63)	.7
Male	8 (42)	14 (37)	
**Cytological diagnosis**			
Bethesda VI (PTC)	13 (68)	30 (79)	.384
Bethesda V (suspicious for PTC)	6 (32)	8 (21)	
**Diabetes mellitus**			
Yes	11 (61)	9 (24)	.008
No	7 (39)	28 (76)	
**BMI (kg/m^2^)**			
Median [IQR]	30.1 [26.5-33.9]	29.43 [25.5-35.5]	.52
**Race**			
White	15 (83)	35 (95)	.14
Asian	2 (11)	1 (2.5)	
Other	1 (6)	1 (2.5)	
**Ethnicity Hispanic**	1 (6)	1 (2.5)	
**Tumor size**			
<10 mm	12 (63)	27 (71)	.876
10-15 mm	7 (37)	11 (29)	
**Time on GLP-1RA (months)**			
Median [IQR]	25 [14-34]		
**Type of GLP-1RA**			
Semaglutide	6 (33)		
Dulaglutide	5 (28)		
Tirzepatide	5 (28)		
Liraglutide	1 (5.5)		
Lixisenatide	1 (5.5)		

Medians and interquartile ranges (IQR) were used to present nonparametric variables.

Abbreviations: BMI, body mass index; GLP-1RA, glucagon-like peptide-1 receptor agonist; IQR, interquartile range; PTC, papillary thyroid carcinoma.

### Changes in Tumor Size During Active Surveillance

**Figure 1.**
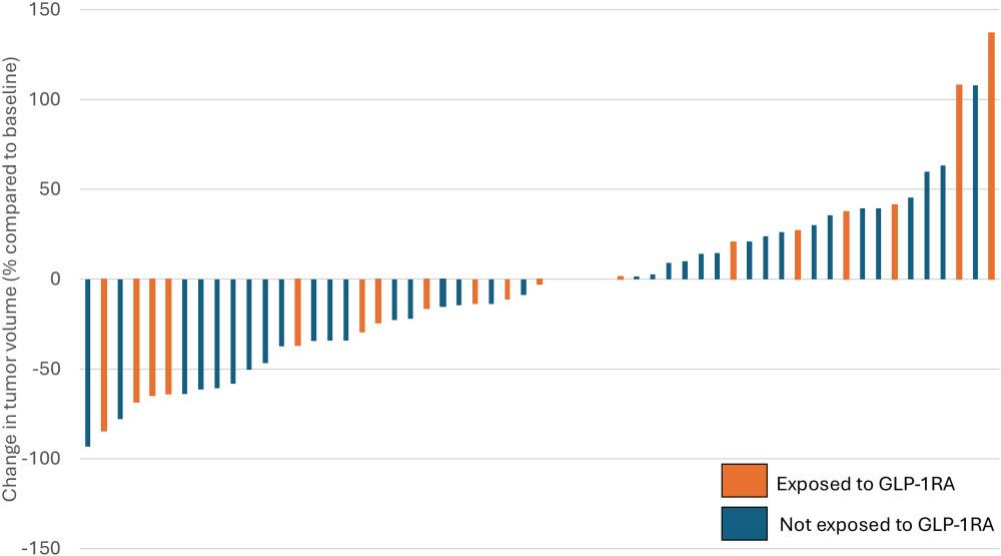
Percent change in tumor volume at final follow-up for all patients exposed and not exposed to GLP-1RA. Waterfall plot showing volume change of 57 carcinomas exposed and not exposed to GLP-1RA during active surveillance. Tumor volume increased >72% in 2 patients exposed to GLP-1RA and 1 patient not exposed to GLP-1RA. Tumor diameter increased by ≥3 mm in 2 patients exposed and 4 patients not exposed to GLP-1RA. Tumor volume decreased >72% in 1 patient exposed and 2 patients not exposed to GLP-1RA. More than 60% volume decrease occurred in 4 patients exposed to GLP-1 and 5 patients not exposed to GLP-1, and any diameter decreased by ≥3 mm in 5 patients exposed to GLP-1RA and 9 patients not exposed to GLP-1RA. Most carcinomas exposed (84.2%) and not exposed (92.1%) to GLP-1RA remained stable. GLP-1RA, glucagon-like peptide-1 receptor agonist.

After a median GLP-1RA exposure of 25 months [IQR: 14-34] and a median follow-up of 5.5 years, there was no statistically significant difference between the proportion of thyroid carcinomas that increased by >72% volume among patients exposed and not exposed to GLP-1RA, 2/19 (10.5%) vs 1/38 (2.6%), *P* = .21, respectively (Figure 1 and Table 2). Moreover, the same proportion of tumors increased any diameter by ≥ 3 mm among patients exposed and not exposed to GLP-1RA (2/19 [10.5%] vs 4/38 [10.5%], *P* = 1, respectively) (Table 2). Two PTCs exposed to GLP-1RA had a significant volume increase of more than 72%. One patient whose tumor increased from 842 to 1197 mm^3^ (+137%) had been on GLP-1RA for years and despite stopping the drug after 2 years on active surveillance (AS), the carcinoma continued to grow off GLP-1RA therapy. Another patient had an early growth of the carcinoma before the GLP-1RA had been started from 25 to 52 mm^3^ (+108%) and continued to grow on the drug. Importantly, drug exposure did not alter the underlying tumor volume growth kinetics in the tumors that experienced growth.

**Table 2.** Change in tumor size at final follow-up among thyroid carcinomas exposed and not exposed to GLP-1RA

	Exposed to GLP-1RA (carcinomas n = 19)		Not exposed to GLP-1RA (carcinomas n = 38)	
	>72% volume	≥ 3 mm	>72% volume *^a^*	≥ 3 mm *^b^*
Increased	2/19 (10.5%)	2/19 (10.5%)	1/38 (2.6%)	4/38 (10.5%)
Stable	16/19 (84.2%)	12/19 (63.2%)	35/38 (92.1%)	25/38 (65.8%)
Decreased	1/19 (5.3%)	5/19 (26.3%)	2/38 (5.3%)	9/38 (23.7%)

^a^ *P* = .53 > 72% volume change comparison between exposed and nonexposed to GLP-1RA.

^b^ *P* = .97 ≥ 3-mm diameter change comparison between exposed and nonexposed to GLP-1RA.

### Individual Tumor Volume Kinetics

**Figure 2.**
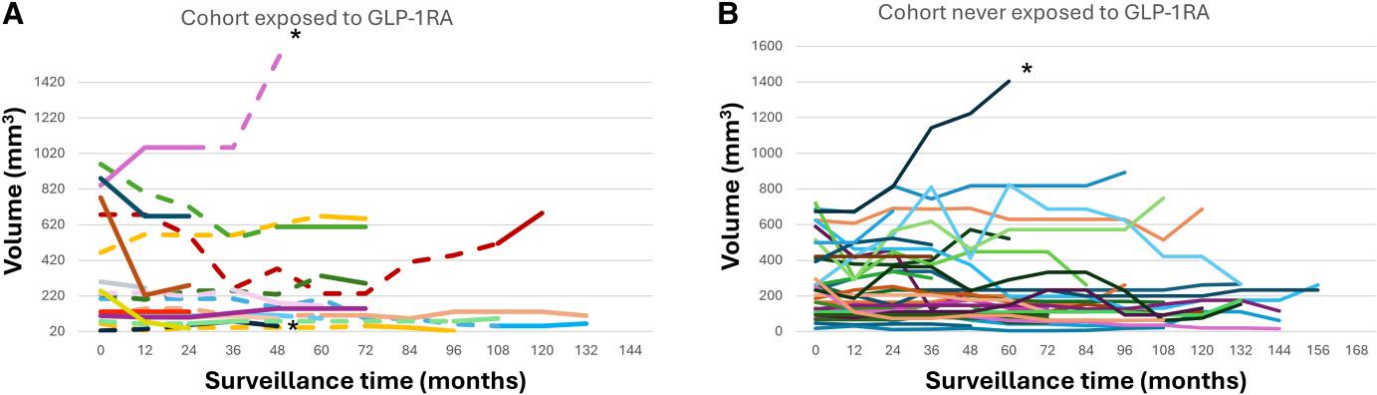
Thyroid carcinoma volume kinetics in patients exposed and not exposed to GLP-1RA. (A) The volumes of 19 carcinomas off (dashed line) and on (solid arrow) GLP-1RA over time represented in the X axis in months. (B) The volumes of 38 carcinomas never exposed to GLP-1RA (solid arrows). *significant volume increase >72%. GLP-1RA, glucagon-like peptide-1 receptor agonist.

Consistent with the expected natural history of low risk PTC during active surveillance, most patients exposed (16/19 [84.2%]) and not exposed (35/38 [92.1%] to GLP-1RA showed “Pattern I-stable,” *P* = .53, when we analyzed tumor volume kinetic patterns as described by Tuttle et al in 2022 [25]) (Fig. 2A and 2B). We did not detect a significant difference in the pattern of tumor volume kinetics among patients exposed and not exposed to GLP-1RA. Two PTCs (10.5%) exposed to GLP-1RA, and 1 (2.6%) PTC not exposed to GLP-1RA showed an early and significant growth of their volume by more than 72%, corresponding to “Pattern II-early increase.” One (5.3%) carcinoma exposed to GLP-1RA followed “Pattern VI-decrease in tumor volume,” from 250 mm^3^ to 39 mm^3^ [−84.4%]) and 2 carcinomas (5.3%) never exposed to GLP-1RA (Figs. 1 and 2) decreased by 93.3% and 78% at final follow-up, *P* = 1.

**Figure 3.**
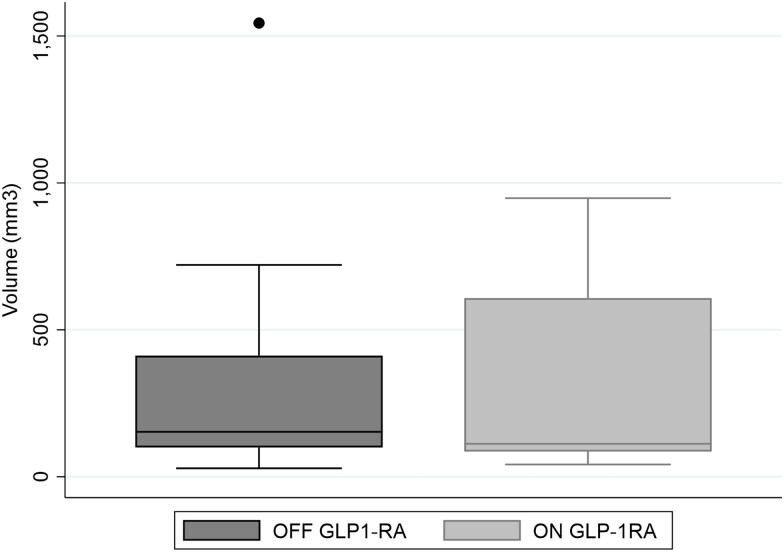
Median volume of carcinomas off and on GLP-1RA in 13 individual patients. Median volume of carcinomas under active surveillance was calculated within 13 patients both off and on GLP-1RA (153 [100-412] vs 112 [86-608] mm^3^, *P* = .97). GLP-1RA, glucagon-like peptide-1 receptor agonist.

In 13 patients who were exposed to GLP-1RA for only part of their microcarcinoma surveillance (median time off GLP-1RA: 57 months, median time on GLP-1RA: 24 months), the median tumor volume off GLP-1RA did not significantly differ from the median volume of the same PTCs on GLP-1RA therapy (153 mm^3^ [100-412] vs 112 mm^3^ [86-608], *P* = .97) (Fig. 3). In the 6 patients who had started GLP-1RA therapy before PTC diagnosis and remained on GLP-1RA after PTC diagnosis, the tumor volume was not significantly different between baseline and final follow up (275 mm^3^ [131-772] at baseline vs 150 mm^3^ [125-280] at final follow up, *P* = .23), although there was a trend toward lower volumes at final follow-up. None of the PTCs exposed to GLP-1RA for the entire AS period (n = 6, 31.6%) had a significant volume increase, most showed stable size over time, and 1 tumor was significantly decreased (−84%, 250 to 39 mm^3^) (Fig. 2A).

**Figure 4.**
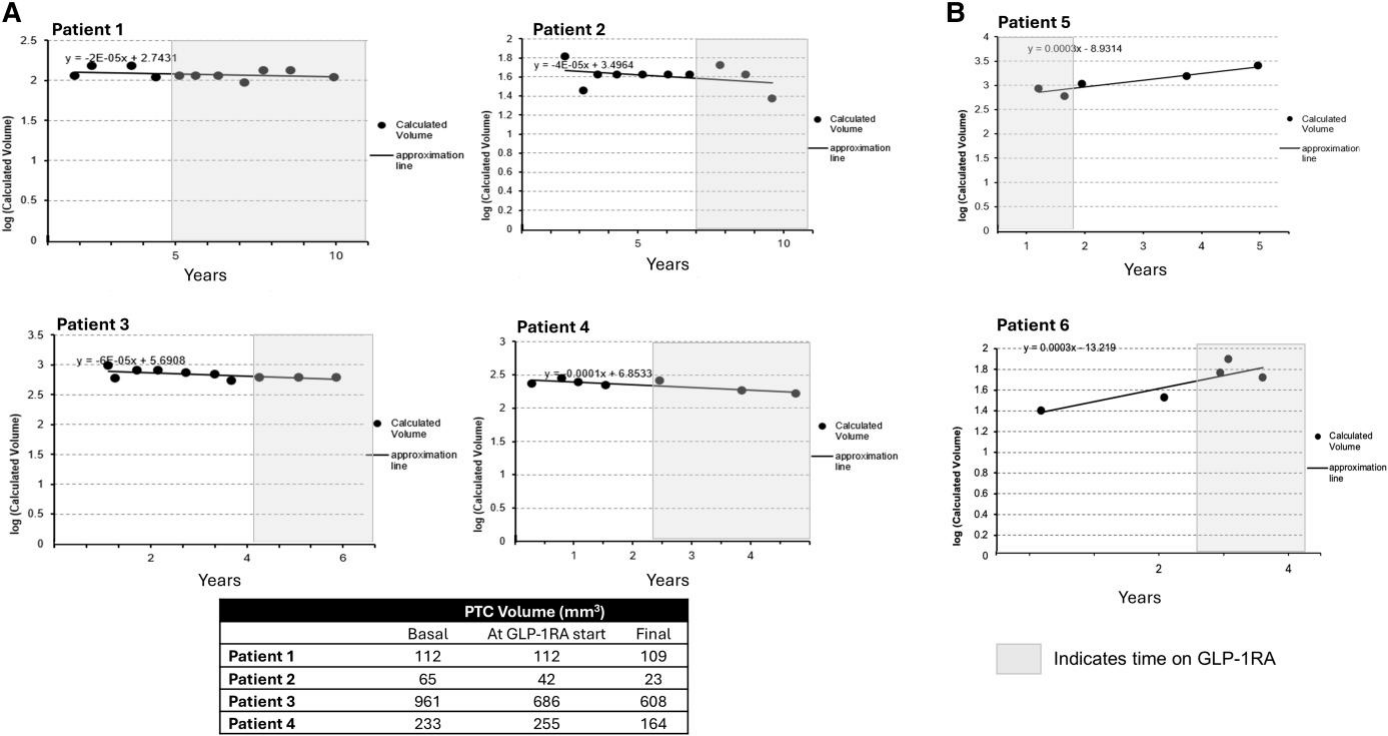
Pattern of growth in representative patients with a measurable TVDT off and on GLP-1RA. (A) Pattern of kinetic growth in 4 patients with sufficient time points to calculate tumor volume doubling time (TVDT) off and on GLP-1RA (shaded area). The x axis shows their time under surveillance in years; the y axis is the log of the calculated volumes. The table describes median volumes off GLP-1RA, at the time of GLP-1RA initiation and at final follow-up. (B) Pattern of growth in patients 5 and 6 with >72% volume increase without evident change in the slope of the growth curve during GLP-1RA exposure. GLP-1RA, glucagon-like peptide-1 receptor agonist; off GLP-1RA, time with no drug exposure; on GLP-1RA, time with drug exposure.

Four patients had at least 3 ultrasounds off and on GLP-1RA therapy that allowed us to calculate the impact of drug exposure on the TVDT. Initiation of GLP-1RA did not significantly change the kinetics of tumor volume over time. Figure 4A shows that TVDT was equivalent off and on the drug and in most cases the tumor volume appeared smaller when the patient was exposed to GLP-1RA. Moreover, in the 2 patients who experienced a significant increase in size of their PTCs during AS (>72% volume), the use of GLP-1RA did not affect the kinetics of tumor growth; that is, the slope of the curve was not changed by drug exposure (Fig. 4B).

**Figure 5.**
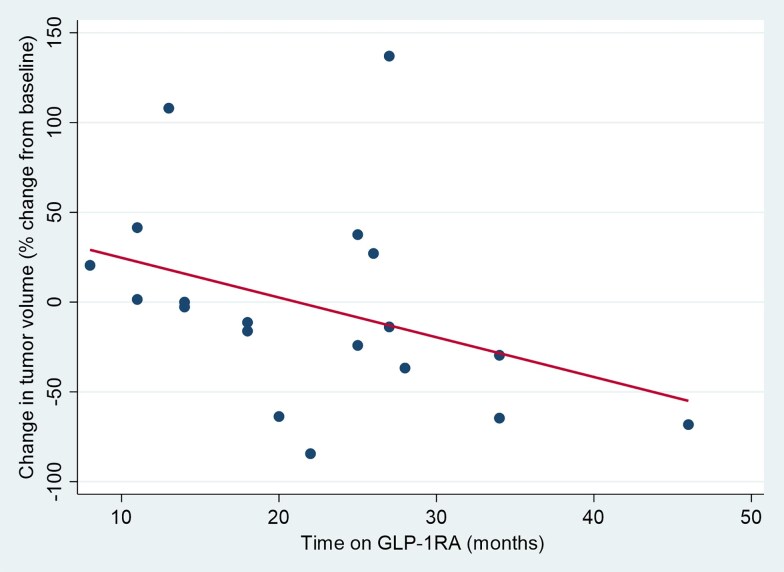
Correlations between time on GLP-1RA and volume change at final follow-up. Scatterplot of time on GLP-1RA in months and final volume change expressed as percent change from baseline with fitted correlation line. GLP-1RA, glucagon-like peptide-1 receptor agonist.

To attempt to find an explanation for size and volume changes over time, we explored whether a longer exposure under GLP-1RA or greater weight loss between presentation and last follow-up was associated with tumor volume changes. Time under GLP-1RA was significantly and inversely correlated with maximal volume change (Spearman rho = −0.51, *P* = .02) (Fig. 5). We did not find a correlation between weight loss (difference in BMI or in weight in kilograms at initial visit and at last follow-up) and maximal volume change (Spearman correlation coefficient rho = 0.17, *P* = .22, and rho = 0.16, *P* = .27, respectively).

At last follow-up, none of the patients had elected a surgical intervention and no regional or distant metastases were detected on any patient during active surveillance.

## Discussion

We report the outcomes of 19 low-risk thyroid carcinomas from 18 patients exposed to GLP-1RA for a median of about 2 years matched 1:2 by BMI and tumor size to 37 patients with 38 low-risk thyroid carcinomas and undergoing active surveillance for more than 5 years. We found that exposure to GLP-1RA was not associated with significant growth of carcinomas or with changes in volume kinetics compared to a controlled group not exposed to GLP-1RA. Within patients off and on GLP-1RA therapy, initiation of the drug did not affect the tumor volume doubling time. Time under GLP-1RA was significantly correlated with carcinoma shrinkage. None of our cases developed regional or distant metastases, consistent with previous reports on PTC under active surveillance from Japan and the United States [25, 26].

Glucagon-like peptide receptor analogs are widely used for the increasing epidemic of obesity and type 2 diabetes mellitus. Beyond their well-documented metabolic benefits and consequent health outcomes for millions of patients, there is growing interest in their potential effects on cancer biology. The role of GLP-1RA in cancer remains complex and not fully understood. He et al reported an elevated GLP-1 receptor expression in 49% of PTC tissues relative to normal thyroid samples but observed no stimulatory effects on cellular proliferation or metabolism following liraglutide and exenatide exposure [27]. In an epidemiologic analysis integrating data from the Thyroid Cancer Genome Atlas project and The Genotype-Tissue Expression repository, increased GLP-1R expression was linked to improved survival in patients with thyroid cancer (hazard ratio: 0.42, logrank *P* = .021) [28]. More recent preclinical studies have demonstrated that liraglutide may inhibit proliferation and migration in papillary thyroid carcinoma cell lines, potentially via PI3K/Akt/mTOR pathway modulation [29]. Similar antiproliferative effects have been described in lung, prostate, and pancreatic cancer models [30-32]. Conversely, a recent retrospective multicenter cohort study that analyzed electronic health records of 113 million US patients found that the risk of thyroid cancer did not differ among patients exposed to GLP-1RA compared to those exposed to insulin, whereas GLP-1RA decreased the risk of 10 other cancers including pancreatic cancer, ovarian cancer, colorectal cancer, and others [33].

In our study, we found that time under GLP-1RA significantly and inversely correlated with volume change at final follow up, but we did not have a sample size large enough to confirm whether weight loss after GLP-1RA use mediated the volume decrease of these low-risk thyroid carcinomas. Excess body weight has been found to significantly increase the risk of thyroid cancer (hazard ratio: 1.33; 95% CI, 1.04-1.70) in a systematic review and meta-analysis of prospective cohort studies [34] and in half a million Asian patients [35]. Additionally, high fasting insulin and insulin resistance have been associated with high prevalence of thyroid carcinoma in a meta-analysis of 14 global case-control studies [36]. The mechanism by which excess body weight increases cancer risk is possibly explained by insulin and IGF. A chronic excess body weight condition increases production of free fatty acids, cytokines such as TNF-α and IL-6, and leptin from adipose tissue, whereas it decreases adiponectin production, which together lead to the development of insulin resistance and chronic hyperinsulinemia [37]. Chronic hyperinsulinemia decreases IGF-binding protein 1 and IGF-binding protein 2 concentrations in blood and other local tissues, which results in an increase in bioavailable free IGF-1. Circulating free IGF-1 increases the risk of many cancer types including colorectal, prostate, and breast cancer. GLP-1RA decrease chronic hyperinsulinemia and an IGF-1 mechanism could possibly mediate the beneficial oncologic effects that we observed with GLP-1RA in this study. Undoubtedly, these findings warrant further investigation to elucidate the role of GLP-1 signaling in carcinogenesis and tumor progression, particularly within thyroid malignancies.

Recent observational studies, both single- and multination registry-based, have explored the incidence of newly diagnosed thyroid cancer in relation to GLP-1RA exposure, yielding conflicting results. Across these investigations, the average duration of drug exposure ranged from 1 to 4 years [11, 14, 15]. Our cohort had a median duration of GLP-1RA exposure exceeding 2 years within a clinical framework of preexisting malignancy under active surveillance. This differs significantly from conventional pharmacoepidemiologic studies on drug-induced de novo carcinogenesis where issues such as cancer latency and lag time are more relevant [38]. We observed that longer duration of GLP-1RA exposure was associated with greater tumor volume decrease. Extended follow-up of a larger cohort is necessary to confirm these preliminary results.

The current clinical study is the first one to report the effect of GLP-1RAs on patients with biopsy-confirmed thyroid cancer undergoing active surveillance. Our findings suggest a safe profile for the use of these agents in a population with low-risk thyroid carcinomas but should be interpreted in the context of several limitations. First, our small sample size and the retrospective nature of our study prevent us from deriving any causal associations between GLP-1RA and tumor kinetics. In this sense, it is plausible that a larger sample of carcinomas undergoing active surveillance may reveal different growth patterns when exposed to GLP-1RA or that the tumor size changes observed in this study may be related to other patient or tumor characteristics independent of GLP-1RA use. We believe that the limited number of patients involved in our cohort could be related to the hesitancy of clinicians to prescribe GLP-1RA to patients with a history of thyroid cancer, particularly to those with active PTC in light of the ongoing debate on the association between GLP-1RA use and risk of thyroid cancer [39]. However, we find the pattern of tumor growth in this small sample comparable to that of patients with microcarcinomas undergoing AS and not exposed to GLP-1RA. Moreover, exposure to the drug did not change the underlying tumor volume kinetics. Second, although single-center data collection may limit generalizability of our findings, the study was conducted within a specialized academic center with a robust active surveillance program for thyroid carcinomas that includes consistent ultrasound assessments by experienced radiologists, all factors that enhance the reliability of our findings. Finally, we investigated GLP-1RA at the drug class level regardless of administered dose or route, with semaglutide and dulaglutide being the most prescribed drugs in the study. We cannot exclude that individual drugs may have a differential effect on thyroid carcinomas. Future studies may assess the risk of thyroid cancer progression by individual molecules.

## Conclusions

This is the first study to examine the effect of GLP-1RA on patients with thyroid carcinomas undergoing active surveillance. Our findings suggest that GLP-1RA exposure does not promote growth or progression of low-risk PTCs under active surveillance for about 5 years. Time under GLP-1RA was associated with tumor shrinkage. Although exploratory in nature and limited by sample size, this study provides early evidence to support the safety of GLP-1RA therapy in this patient population. Larger and longer cohorts of patients with low-risk thyroid carcinomas exposed to GLP-1RA are needed to confirm our findings. These preliminary results may help inform clinical decision-making and encourage appropriate use of GLP-1RAs among patients with coexisting low-risk thyroid carcinomas, diabetes mellitus and obesity.

## Data Availability

Some or all datasets generated during and/or analyzed during the current study are not publicly available but are available from the corresponding author on reasonable request.

## Abbreviations

AS: active surveillance
BMI: body mass index
FDA: US Food and Drug Administration
GLP-1RA: glucagon-like peptide-1 receptor agonist
IQR: interquartile range
MSKCC: Memorial Sloan Kettering Cancer Center
MTC: medullary thyroid cancer
PTC: papillary thyroid cancer
TVDT: tumor volume doubling time
